# Palliative Versorgung und Therapiezieländerung bei Herzinsuffizienz im ambulanten Bereich: Eine Umfrage zur aktuellen Versorgungswirklichkeit unter niedergelassenen Ärzt:innen in Deutschland

**DOI:** 10.1007/s00059-025-05325-x

**Published:** 2025-06-26

**Authors:** Moritz Blum, Mark Weber-Krüger, Hashim Abdul-Khaliq, Bernd Alt-Epping, Marc Dittrich, Tanja Henking, Gerald Neitzke, Harald Rittger, Henrikje Stanze, Dorit Knappe, Klaus K. Witte, Jochen Dutzmann, Franz Goss

**Affiliations:** 1 Klinik für Thorax‑, Herz- und Gefäßchirurgie, Deutsches Herzzentrum der Charité (DHZC) – Medical Heart Center of Charité and German Heart Institute Berlin, Berlin, Deutschland; 2 Charité – Universitätsmedizin Berlin, corporate member of Freie Universität Berlin and Humboldt-Universität zu Berlin, Berlin, Deutschland; 3 Klinik für Palliativmedizin, Universitätsmedizin Göttingen, Göttingen, Deutschland; 4 Klinik für Kinderkardiologie, Universitätsklinikum des Saarlandes, Homburg (Saar), Deutschland; 5 Klinik für Palliativmedizin, Universitätsklinikum Heidelberg, Heidelberg, Deutschland; 6 Klinik für Innere Medizin 2, Universitätsklinikum Regensburg, Regensburg, Deutschland; 7 Professur für Gesundheits- und Medizinrecht und Strafrecht, Technische Hochschule Würzburg-Schweinfurt, Würzburg, Deutschland; 8 Institut für Ethik, Geschichte und Philosophie der Medizin, Medizinische Hochschule Hannover, Hannover, Deutschland; 9 Klinik für Herz- und Lungenerkrankungen, Klinikum Fürth, Fürth, Deutschland; 10 Professur für Pflegewissenschaft und Palliative Care, Hochschule Bremen, Bremen, Deutschland; 11 Klinik für Kardiologie und internistische Intensivmedizin, Asklepios Klinikum St. Georg, Hamburg, Deutschland; 12 Leeds Institute of Cardiovascular and Metabolic Medicine, University of Leeds, Leeds, Großbritannien; 13 Klinik für Innere Medizin III (Kardiologie, Angiologie und Internistische Intensivmedizin), Universitätsklinikum Halle (Saale), Ernst-Grube-Str. 40, 06120 Halle (Saale), Deutschland; 14 Bundesverband Niedergelassener Kardiologen e. V., München, Deutschland

**Keywords:** Herzinsuffizienz, Palliativmedizin, Ambulante Versorgung, Hausärzte, Niedergelassene Kardiologen, Heart failure, Palliative medicine, Outpatient care, General practitioners, Office-based cardiologists

## Abstract

**Hintergrund:**

Palliative Versorgung ist Teil der ganzheitlichen Behandlung bei fortgeschrittener Herzinsuffizienz. Wie diese Versorgung im ambulanten Bereich in Deutschland derzeit tatsächlich aussieht, ist unklar.

**Methode:**

Wir führten eine Umfrage zur aktuellen palliativen Versorgungspraxis bei fortgeschrittener Herzinsuffizienz unter niedergelassenen Kardiolog:innen und Hausärzt:innen in Deutschland durch. Die Umfrage wurde von einer multiprofessionellen Projektgruppe der Deutschen Gesellschaft für Kardiologie – Herz- und Kreislaufforschung e. V. entwickelt und durch den Bundesverband Niedergelassener Kardiologen e. V. online durchgeführt.

**Ergebnisse:**

An der Studie nahmen 235 Personen teil. Eine Mehrheit der Befragten gab an, häufig oder immer mit Patient:innen mit fortgeschrittener Herzinsuffizienz über ihre Therapieziele gesprochen zu haben. Hausärzt:innen gaben wesentlich öfter als Kardiolog:innen an, immer oder häufig Aufgaben der palliativen Grundversorgung zu übernehmen. Keine:r der befragten niedergelassenen Kardiolog:innen, jedoch 35,1 % der Hausärzt:innen gaben an, bei Patient:innen mit fortgeschrittener Herzinsuffizienz häufig oder immer eine spezialisierte ambulante Palliativversorgung (SAPV) verordnet zu haben. Über 90 % der Kardiolog:innen gaben an, Patient:innen selten oder nie weiter zu betreuen, wenn diese nicht mehr selbst in die Praxis kommen konnten.

**Konklusion:**

Nur wenige Niedergelassene in Deutschland überweisen ihre Patient:innen mit fortgeschrittener Herzinsuffizienz an spezialisierte palliativmedizinische Versorgungsstrukturen. Palliative Grundversorgung und die Involvierung der SAPV werden wesentlich häufiger von Hausärzt:innen als von niedergelassen Kardiolog:innen übernommen.

## Abstract (en)

**Background:**

Palliative care is a crucial part of the holistic management of advanced heart failure; however, it remains unclear how palliative care is currently provided in the outpatient sector in Germany.

**Methods:**

We conducted a survey among office-based cardiologists and general practitioners (GPs) in Germany on the current provision of palliative care for people with advanced heart failure. The survey was developed by a multiprofessional project group of the German Cardiac Society (DGK e. V.) and administered online by the National Association of Office-Based Cardiologists (BNK e.V.).

**Results:**

A total of 235 individuals participated in the study. The majority of respondents reported frequently or always discussing goals of care with patients with advanced heart failure. The GPs reported significantly more often than cardiologists that they always or frequently address primary palliative care needs. None of the surveyed office-based cardiologists but 35.1% of GPs stated that they frequently or always prescribe specialized outpatient palliative care (SAPV) for patients with advanced heart failure. Over 90% of the cardiologists reported that they rarely or never continue to care for patients when they cannot come to the practice themselves.

**Conclusion:**

Only few office-based physicians in Germany regularly refer patients with advanced heart failure to specialized palliative care services. Primary palliative care and the involvement of SAPV are significantly more often managed by GPs than by office-based cardiologists.

## Einleitung

Palliative Versorgung kann durch multiprofessionelles Management körperlicher, psychischer, sozialer und spiritueller Probleme die Lebensqualität von Menschen mit schweren Erkrankungen verbessern. Idealerweise sollte sie frühzeitig im Krankheitsverlauf beginnen, begleitend zur lebensverlängernden Therapie [1, 2]. Die positiven Effekte von palliativer Versorgung auf die Lebensqualität sind dabei nicht nur für Patient:innen mit Tumorerkrankungen, sondern auch für solche mit nichtmalignen Erkrankungen nachgewiesen [3].

Dementsprechend betrachten Herzinsuffizienzleitlinien palliative Versorgung auch als einen wichtigen Bestandteil einer ganzheitlichen Behandlung [4]. Alle Gesundheitsdienstleistenden, die Patient:innen mit Herzinsuffizienz behandeln, sollten zur palliativen Grundversorgung beitragen, z. B. durch ganzheitliches Assessment und Management körperlicher Symptome und psychosozialer Probleme sowie durch empathische Kommunikation über Prognose und Therapieziele. Bei schwerer Symptomlast und komplexen Problemen sollten Patient:innen an spezialisierte palliativmedizinische und -pflegerische Versorgungsangebote weitervermittelt werden. Die meisten Patient:innen mit Herzinsuffizienz in Deutschland werden im ambulanten Sektor betreut. Für die palliative Grundversorgung sind Hausärzt:innen und niedergelassene Kardiolog:innen zuständig, bei komplexem Unterstützungsbedarf kommen ambulante Einrichtungen der spezialisierten ambulanten Palliativversorgung (SAPV) zum Tragen [5–7].

Die tatsächliche palliative Versorgungssituation von Patient:innen mit Herzinsuffizienz im ambulanten Bereich ist bislang unzureichend charakterisiert [7]. In welchem Umfang Niedergelassene selbst palliative Grundversorgung leisten und wie sie mit der SAPV zusammenarbeiten, wurde unseres Wissens noch nicht systematisch untersucht. Als ersten Schritt zu einer besseren Erfassung führten wir deshalb eine Umfragestudie unter niedergelassenen Kardiolog:innen und Hausärzt:innen in Deutschland zur palliativen Versorgungspraxis bei fortgeschrittener Herzinsuffizienz durch.

**Abb. 1:**
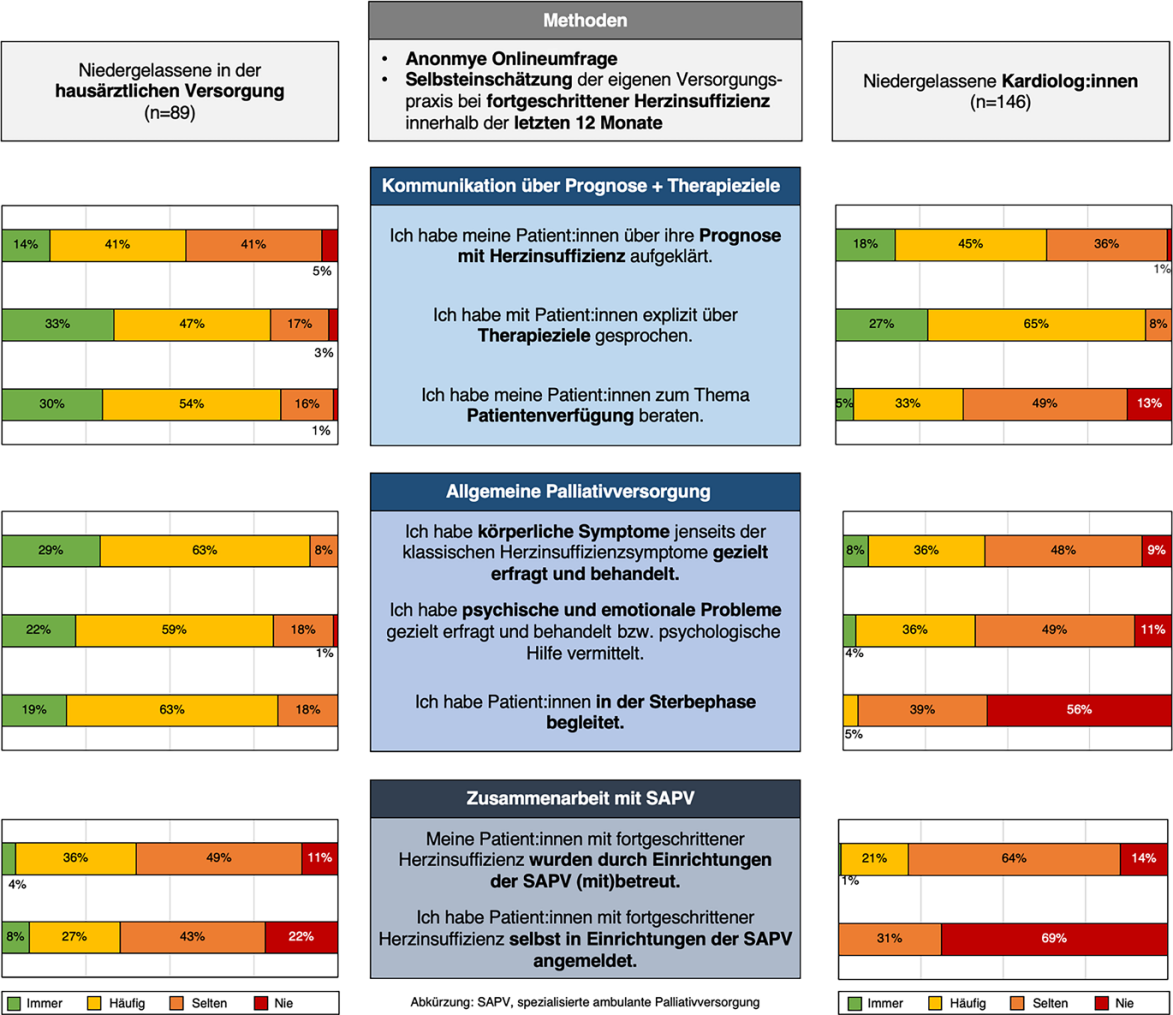
Grafische Zusammenfassung (*SAPV* spezialisierte ambulante Palliativversorgung)

## Methoden

### Regulatorische Aspekte

Die vorliegende Fragebogenstudie wurde durch Mitglieder der Projektgruppe „Ethik in der Kardiologie“ der Deutschen Gesellschaft für Kardiologie – Herz-Kreislauf-Forschung e. V. konzipiert und durchgeführt. Als anonyme Umfrage ohne Beteiligung von Patient:innen war diese Studie nicht zustimmungspflichtig durch eine Ethikkommission.

### Fragebogenentwicklung

Zwischen April und Juli 2024 entwickelte die Projektgruppe, bestehend aus Expert:innen aus ambulanter und stationärer Kardiologie, Palliativmedizin, Pflegewissenschaften, klinischer Ethik und Rechtswissenschaften, einen Fragebogen zur Erfassung der palliativen Versorgungspraxis von niedergelassenen Kardiolog:innen und Hausärzt:innen in Deutschland. Zur Quantifizierung der Zustimmung zu einzelnen Frage-Items wählten wir 4‑Punkt-Likert-Skalen, um der Tendenz zur Mitte entgegenzuwirken. Der Fragebogen wurde multiprofessionell und interdisziplinär auf Augenscheinvalidität überprüft und in 3 Runden kollaborativ überarbeitet.

### Fragebogendurchführung

Der Fragebogen wurde in Zusammenarbeit mit dem Bundesverband Niedergelassener Kardiologen e. V. (BNK) auf der Umfrageplattform surveymonkey.com (SurveyMonkey Europe UC, Dublin, Irland) als anonyme Onlineumfrage ausgestaltet. Die Onlineumfrage war vom 23.08.2024 bis zum 01.12.2024 geöffnet und wurde über E‑Mail-Verteiler des BNK sowie von MEDI Baden-Württemberg e.V., einem Verein niedergelassener Ärzt:innen aller Fachrichtungen, versendet. Von 1199 kontaktierten niedergelassenen Kardiolog:innen nahmen 146 (Antwortrate: 12,2 %) und von etwa 1000 kontaktierten Hausärzt:innen nahmen 89 an der Umfrage teil (Antwortrate: ca. 8,9 %).

### Fragebogenauswertung

Die statistische Auswertung der Umfrageantworten wurde mit R: A Language and Environment for Statistical Computing, Version 4.2.2 (2022-10-31; R Foundation for Statistical Computing, Vienna, Austria) durchgeführt [8]. Die Antworten auf 4‑Punkt-Likert-Skalen wurden als kategorielle Variable ausgewertet und als absolute Anzahl und Prozentsatz von der Summe aller auswertbarer Antworten angegeben. Für eine Subgruppenanalyse stratifizierten wir die Studienpopulation in niedergelassene Kardiolog:innen (Fachärzt:innen für Kardiologie in Niederlassung) und Niedergelassene in der hausärztlichen Versorgung (Fachärzt:innen für Allgemeinmedizin und Fachärzt:innen für Innere Medizin in der hausärztlichen Versorgung; kurz: Hausärzt:innen). Unterschiede zwischen den Subgruppen wurden mit dem Chi-Quadrat-Test auf ihre statistische Signifikanz überprüft. Als Signifikanzniveau setzten wir ein α von 0,05 fest.

## Ergebnisse

### Studienpopulation

Insgesamt begannen 235 Teilnehmende die Onlineumfrage, 205 (87,2 %) beantworteten die Umfrage vollständig. Der Großteil der Teilnehmenden war männlich (74,6 %), über 50 Jahre alt (76 %) und bereits seit mehr als 20 Jahren niedergelassen (45,3 %). Niedergelassene Kardiolog:innen machten 62,1 % der Population aus, Niedergelassene in der hausärztlichen Versorgung 37,9 %. Die Zusatzbezeichnung Palliativmedizin besaßen 30,3 % der Hausärzt:innen und 2,1 % der niedergelassenen Kardiolog:innen (*p* < 0,001). Weitere Informationen zur Studienpopulation finden sich in Tab. 1.

**Tab. 1:** Charakteristika der Studienpopulation

	Insgesamt	Niedergelassene in der hausärzlichen Versorgung	Niedergelassene in der Kardiologie	*p*
	*n* = 235	*n* = 89	*n* = 146	
Facharzt – *n* (%)				< 0,001
– Fachärzt:in für Allgemeinmedizin	74 (31,5)	74 (83,1)	0 (0,0)	–
– Fachärzt:in für Innere Medizin	15 (6,4)	15 (16,9)	0 (0,0)	
– Fachärzt:in für Kardiologie	146 (62,1)	0 (0,0)	146 (100,0)	
Geschlecht weiblich – *n* (%)	59 (25,4)	30 (34,1)	29 (20,1)	0,027
Alter – *n* (%)				0,234
– < 40 Jahre	6 (2,6)	2 (2,2)	4 (2,8)	–
– 40–50 Jahre	50 (21,4)	17 (19,1)	33 (22,8)	
– 50–60 Jahre	82 (35,0)	26 (29,2)	56 (38,6)	
– > 60 Jahre	96 (41,0)	44 (49,4)	52 (35,9)	
Bundesland				< 0,001
– Baden-Württemberg	123 (52,3)	89 (100,0)	34 (23,3)	–
– Nordrhein	31 (13,2)	0 (0,0)	31 (21,2)	
– Niedersachsen	17 (7,2)	0 (0,0)	17 (11,6)	
– Bayern	15 (6,4)	0 (0,0)	15 (10,3)	
– andere Bundesländer	49 (20,9)	0 (0,0)	49 (33,6)	
Niedergelassen seit (%)				< 0,001
– < 5 Jahren	29 (12,4)	7 (8,0)	22 (15,1)	–
– 5–10 Jahren	33 (14,1)	8 (9,1)	25 (17,1)	
– 11–20 Jahren	66 (28,2)	17 (19,3)	49 (33,6)	
– > 20 Jahren	106 (45,3)	56 (63,6)	50 (34,2)	
Zusatzbezeichnung Palliativmedizin	30 (12,8)	27 (30,3)	3 (2,1)	< 0,001
Ich habe Patient:innen mit fortgeschrittener Herzinsuffizienz behandelt, d. h. Patient:innen, die trotz optimaler Herzinsuffizienztherapie schwere Symptome hatten und dadurch in ihrem Alltag eingeschränkt waren bzw. wiederholt hospitalisiert werden mussten				
– immer	29 (12,7)	14 (16,7)	15 (10,4)	< 0,001
– häufig	127 (55,7)	34 (40,5)	93 (64,6)	
– selten	72 (31,6)	36 (42,9)	36 (25,0)	
– nie	0 (0,0)	0 (0,0)	0 (0,0)	
Ich habe Patient:innen weiterbetreut, obwohl diese nicht mehr selbst in die Praxis kommen konnten				
– immer	22 (9,6)	21 (24,7)	1 (0,7)	< 0,001
– häufig	46 (20,1)	41 (48,2)	5 (3,5)	
– selten	70 (30,6)	22 (25,9)	48 (33,3)	
– nie	91 (39,7)	1 (1,2)	90 (62,5)	
Auf welche Weise? (Mehrfachauswahl möglich)				
– telefonische ärztliche Beratung	112 (81,8)	64 (76,2)	48 (90,6)	0,058
– eigene Hausbesuche während der regulären Sprechstundenzeit	78 (56,9)	68 (81,0)	10 (18,9)	< 0,001
– eigene Hausbesuche bei Notfällen nachts/am Wochenende	46 (33,6)	32 (38,1)	14 (26,4)	0,221
– Beratung von Hausärzt:innen	44 (32,1)	6 (7,1)	38 (71,7)	< 0,001

Die Kategorie „Niedergelassene in der hausärztlichen Versorgung“ beinhaltet Fachärzt:innen für Allgemeinmedizin und Fachärzt:innen für Innere Medizin in der hausärztlichen Versorgung. Subgruppen wurden mittels Chi-Quadrat-Test verglichen

### Erfahrung mit der Versorgung bei schwerer Herzinsuffizienz

Eine graphische Zusammenfassung der wichtigsten Ergebnisse bietet Abb. 1. Alle Befragten gaben an, in den letzten 12 Monaten Patient:innen mit schwerer Herzinsuffizienz, d. h. solche mit einschränkenden Symptomen trotz optimaler Therapie und wiederholten Hospitalisierungen, behandelt zu haben; 68,4 % behandelten solche Patient:innen häufig oder immer (Hausärzt:innen: 57,1 %, Kardiolog:innen: 75 %; *p* < 0,001). Eine ischämische Herzerkrankung wurde mit 89,9 % am häufigsten als zugrunde liegende Erkrankung genannt.

Der Großteil der niedergelassenen Kardiolog:innen gab an, Patient:innen nie (62,5 %) oder selten (33,3.%) weiterzubehandeln, wenn diese nicht mehr in der Lage waren, in die Praxis zu kommen. Die Kardiolog:innen, die Patient:innen auch dann weiterbehandelten, leisteten v. a. telefonische Betreuung (90,6 %). Hausärzt:innen gaben an, an die Wohnung gebundene Herzinsuffizienzpatient:innen häufig (48,2 %) oder immer (24,7 %) weiterzubehandeln, meistens durch Hausbesuche während der regulären Sprechzeiten (81,0 %), seltener auch notfallmäßig nachts oder am Wochenende (38,1 %).

### Kommunikation über Prognose und Therapieziele

Etwas mehr als die Hälfte der Befragten gab an, in den letzten 12 Monaten häufig (43,4 %) oder immer (16,4 %) mit ihren Patient:innen darüber gesprochen zu haben, dass Herzinsuffizienz eine unheilbare Erkrankung ist, die chronisch-progressiv verläuft und bei vielen Menschen zum vorzeitigen Tod führt. Als Anlässe für solche Gespräche wurden am häufigsten wiederholte Krankenhauseinweisungen (82,2 %) und eine Verschlechterung der Herzinsuffizienzsymptomatik (77,8 %) angegeben. Die Erstdiagnose einer Herzinsuffizienz wurde nur selten (31,9 %) als Anlass genannt, über die Prognose und den weiteren Krankheitsverlauf zu sprechen.

Knapp drei Viertel der befragten Ärzt:innen sprachen außerdem häufig (57,1 %) oder immer (13,8 %) mit Herzinsuffizienzpatient:innen über Therapieziele und Therapiezieländerungen (Tab. 2). Häufigster Anlass für solche Gespräche waren der eigene subjektive klinische Eindruck (92,3 %) oder akute Ereignisse (65,1 %). Oft fanden sie auch auf Initiative von Patient:innen (57,4 %) oder Angehörigen (53,6 %) statt. Inhaltlich ging es in den Gesprächen häufig um Entscheidungen bezüglich zukünftiger interventioneller Eingriffe (83,2 %), zukünftiger Operationen (81,7 %), zukünftiger Intensivbehandlungen (78,8 %) oder zukünftiger Krankenhauseinweisungen (70,7 %). Etwa die Hälfte der Befragten gab an, häufig oder immer Beratungsgespräche zum Thema Patientenverfügung (54,0 %) oder Vorsorgevollmacht (52,4 %) durchgeführt zu haben. Hausärzt:innen berieten ihre Patienten signifikant häufiger zu den Themen Patientenverfügung und Vorsorgevollmacht als Kardiolog:innen (*p* < 0,001). Ansonsten zeigten sich kaum relevante Unterschiede zwischen den beiden Subgruppen bezüglicher der Kommunikation über Prognose und Therapieziele (Tab. 2).

**Tab. 2:** Kommunikation über Prognose und Therapieziele

	Insgesamt	Niedergelassene in der hausärztlichen Versorgung	Niedergelassene in der Kardiologie	*p*
	*n* = 235	*n* = 89	*n* = 146	
Ich habe mit meinen Patient:innen besprochen, dass die Herzinsuffizienz eine unheilbare Erkrankung ist, die chronisch-progressiv verläuft und bei vielen Menschen zum vorzeitigen Tod führt				
– immer	37 (16,4)	12 (14,3)	25 (17,6)	0,371
– häufig	98 (43,4)	34 (40,5)	64 (45,1)	
– selten	85 (37,6)	34 (40,5)	51 (35,9)	
– nie	6 (2,7)	4 (4,8)	2 (1,4)	
Was waren für Sie Anlässe, über diese Thematik zu sprechen? (Mehrfachauswahl möglich)				
– Wiederholte Krankenhauseinweisungen innerhalb 6 Monate	111 (82,2)	42 (93,3)	69 (76,7)	0,032
– subjektive Verschlechterung der Herzinsuffizienzsymptomatik	105 (77,8)	37 (82,2)	68 (75,6)	0,51
– objektive Verschlechterung hämodynamischer Parameter	92 (68,1)	31 (68,9)	61 (67,8)	1
– zunehmende Gebrechlichkeit (Stürze, beginnende Demenz)	84 (62,2)	32 (71,1)	52 (57,8)	0,188
– Indikation ICD/CRT	79 (58,5)	22 (48,9)	57 (63,3)	0,155
– Neudiagnose oder Progress relevanter Komorbiditäten	47 (34,8)	15 (33,3)	32 (35,6)	0,949
– Erstdiagnose einer Herzinsuffizienz	43 (31,9)	9 (20,0)	34 (37,8)	0,058
Gab es Gründe, die Sie davon abgehalten haben? (Mehrfachauswahl möglich)				
– fehlende Zeitressourcen	35 (44,3)	7 (25,0)	28 (54,9)	0,02
– Sorge vor Nocebo-Effekten	27 (34,2)	5 (17,9)	22 (43,1)	0,044
– Ich fühle mich in der Gesprächsführung unsicher	4 (5,1)	1 (3,6)	3 (5,9)	1
Ich habe mit meinen kardiologischen Patient:innen explizit über Therapieziele gesprochen (z. B. Lebenszeitverlängerung, Vermeidung von Krankenhausaufenthalten, Symptomverbesserung)				
– immer	63 (29,4)	25 (33,3)	38 (27,3)	0,012
– häufig	125 (58,4)	35 (46,7)	90 (64,7)	
– selten	24 (11,2)	13 (17,3)	11 (7,9)	
– nie	2 (0,9)	2 (2,7)	0 (0,0)	
Ich habe mit meinen kardiologischen Patient:innen über Therapiezieländerungen (z. B. Verzicht auf Krankenhauseinweisungen, Beendigung lebensverlängernder Therapien, z. B. ICD, LVAD, Verzicht auf Eingriffe, Zulassen eines Versterbens) gesprochen				
– immer	30 (13,8)	16 (20,5)	14 (10,1)	0,206
– häufig	124 (57,1)	41 (52,6)	83 (59,7)	
– selten	60 (27,6)	20 (25,6)	40 (28,8)	
– nie	3 (1,4)	1 (1,3)	2 (1,4)	
In welchen Situationen? (Mehrfachauswahl möglich)				
– aufgrund meines eigenen subjektiven klinischen Eindrucks	193 (92,3)	69 (90,8)	124 (93,2)	0,712
– nach akuten Ereignissen	136 (65,1)	56 (73,7)	80 (60,2)	0,068
– auf Initiative der Patientin/des Patienten	120 (57,4)	43 (56,6)	77 (57,9)	0,968
– auf Initative von Angehörigen	112 (53,6)	40 (52,6)	72 (54,1)	0,948
– auf Initiativen von Mitbehandelnden	40 (19,1)	8 (10,5)	32 (24,1)	0,027
Über folgende Dinge habe ich mit Patient:innen gesprochen: (Mehrfachauswahl möglich)				
– Entscheidung gegen kardiologische Interventionen in der Zukunft	173 (83,2)	59 (77,6)	114 (86,4)	0,153
– Entscheidung gegen operative Eingriffe in der Zukunft	170 (81,7)	55 (72,4)	115 (87,1)	0,014
– Entscheidung gegen Intensivbehandlung in der Zukunft	164 (78,8)	68 (89,5)	96 (72,7)	0,008
– Entscheidung gegen Krankenhauseinweisungen in der Zukunft	147 (70,7)	66 (86,8)	81 (61,4)	< 0,001
– Entscheidung gegen Organersatzverfahren in der Zukunft	115 (55,3)	40 (52,6)	75 (56,8)	0,66
– Beendigung von Device-Therapien	110 (52,9)	22 (28,9)	88 (66,7)	< 0,001
– Beendigung medikamentöser Therapien	78 (37,5)	39 (51,3)	39 (29,5)	0,003
Ich habe Patient:innen mit fortgeschrittener Herzinsuffizienz zum Thema Patientenverfügung beraten				
– immer	30 (14,2)	23 (30,3)	7 (5,2)	< 0,001
– häufig	84 (39,8)	40 (52,6)	44 (32,6)	
– selten	78 (37,0)	12 (15,8)	66 (48,9)	
– nie	19 (9,0)	1 (1,3)	18 (13,3)	
Ich habe Patient:innen mit fortgeschrittener Herzinsuffizienz zum Thema Vorsorgevollmacht beraten				
– immer	26 (12,4)	21 (27,6)	5 (3,7)	< 0,001
– häufig	84 (40,0)	39 (51,3)	45 (33,6)	
– selten	74 (35,2)	14 (18,4)	60 (44,8)	
– nie	26 (12,4)	2 (2,6)	24 (17,9)	
Ich habe mich bei ethisch konfliktreichen Therapieentscheidungen mit Kolleg:innen ausgetauscht				
– immer	10 (4,7)	3 (3,9)	7 (5,2)	0,088
– häufig	80 (37,7)	21 (27,3)	59 (43,7)	
– selten	104 (49,1)	46 (59,7)	58 (43,0)	
– nie	18 (8,5)	7 (9,1)	11 (8,1)	
Auf welche Art und Weise (Mehrfachauswahl möglich)				
– kollegialer Austausch in meiner Praxis	117 (61,3)	36 (52,2)	81 (66,4)	0,075
– kollegialer Austausch außerhalb der Praxis (z. B. Qualitätszirkel)	105 (55,0)	49 (71,0)	56 (45,9)	0,001
– Austausch mit Hausärzt:innen	103 (53,9)	14 (20,3)	89 (73,0)	< 0,001
– Ambulante Ethikberatung	3 (1,6)	3 (4,3)	0 (0,0)	0,086

Die Kategorie „Niedergelassene in der hausärztlichen Versorgung“ beinhaltet Fachärzt:innen für Allgemeinmedizin und Fachärzt:innen für Innere Medizin in der hausärztlichen Versorgung. Subgruppen wurden mittels Chi-Quadrat-Test verglichen

*CRT* „cardiac resynchronization therapy“, *ICD* implantierbarer Kardioverter-Defibrillator, *LVAD* „left ventricular assist device“

### Palliative Grundversorgung durch Niedergelassene

Der Großteil der befragen Hausärzt:innen gab an, häufig (70,3 %) oder immer (12,2 %) allgemeine Palliativversorgung bei Herzinsuffizienz geleistet zu haben. Konkreter gefragt, gab die Mehrheit der Hausärzt:innen an, häufig (57,5 %) oder immer (9,6 %) therapierefraktäre Dyspnoe mit Opioiden behandelt zu haben, häufig (62,5 %) oder immer (29,2 %) körperliche Beschwerden jenseits der klassischen Herzinsuffizienzsymptome erfragt und behandelt zu haben, häufig (58,9 %) oder immer (21,9 %) psychische Probleme erfragt und behandelt zu haben, häufig (61,6 %) oder immer (32,9 %) Patient:innen bei der Versorgungsplanung unterstützt zu haben sowie häufig (63,0 %) oder immer (19,2 %) Herzinsuffizienzpatient:innen in der Sterbephase begleitet zu haben.

Niedergelassene Kardiolog:innen hingegen gaben größtenteils an, selten (49,2 %) oder nie (43,2 %) selbst allgemeine Palliativversorgung geleistet zu haben. Körperliches und psychisches Symptommanagement, Opioidtherapie bei Herzinsuffizienz, Unterstützung bei der Organisation der häuslichen Versorgung und Sterbebegleitung leistete der Großteil der Kardiolog:innen selten oder nie (Tab. 3). Allerdings gaben 70,9 % der niedergelassenen Kardiolog:innen an, implantierte Defibrillatoren deaktiviert zu haben. Im seltenen Fall der Deaktivierung war der häufigste Grund der ausdrückliche Wunsch der Patient:innen (82,7 %).

**Tab. 3:** Eigenes palliatives Handeln durch Niedergelassene

	Insgesamt	Niedergelassene in der hausärztlichen Versorgung	Niedergelassene in der Kardiologie	*p*
	*n* = 235	*n* = 89	*n* = 146	
Ich habe selbst bei meinen Patient:innen allgemeine Palliativversorgung geleistet				
– immer	9 (4,4)	9 (12,2)	0 (0,0)	< 0,001
– häufig	62 (30,1)	52 (70,3)	10 (7,6)	
– selten	76 (36,9)	11 (14,9)	65 (49,2)	
– nie	59 (28,6)	2 (2,7)	57 (43,2)	
Ich habe Dyspnoe, die nicht auf krankheitsmodifizierende Herzinsuffizienztherapien anspricht (z. B. durch Opioide) behandelt				
– immer	7 (3,4)	7 (9,6)	0 (0,0)	< 0,001
– häufig	53 (25,9)	42 (57,5)	11 (8,3)	
– selten	77 (37,6)	24 (32,9)	53 (40,2)	
– nie	68 (33,2)	0 (0,0)	68 (51,5)	
Ich habe andere körperliche Symptome jenseits der klassischen Herzinsuffizienzsymptome gezielt erfragt und behandelt				
– immer	31 (15,2)	21 (29,2)	10 (7,6)	< 0,001
– häufig	92 (45,1)	45 (62,5)	47 (35,6)	
– selten	69 (33,8)	6 (8,3)	63 (47,7)	
– nie	12 (5,9)	0 (0,0)	12 (9,1)	
Ich habe psychische und emotionale Probleme gezielt erfragt und behandelt bzw. psychologische/psychiatrische Hilfe vermittelt				
– immer	21 (10,2)	16 (21,9)	5 (3,8)	< 0,001
– häufig	91 (44,4)	43 (58,9)	48 (36,4)	
– selten	77 (37,6)	13 (17,8)	64 (48,5)	
– nie	16 (7,8)	1 (1,4)	15 (11,4)	
Ich habe Patient:innen bei der Versorgungsplanung unterstützt (z. B. durch Vermittlung von Pflegediensten, Hilfsmitteln etc.)				
– immer	24 (11,7)	24 (32,9)	0 (0,0)	< 0,001
– häufig	68 (33,2)	45 (61,6)	23 (17,4)	
– selten	72 (35,1)	4 (5,5)	68 (51,5)	
– nie	41 (20,0)	0 (0,0)	41 (31,1)	
Ich habe Patient:innen in der Sterbephase begleitet				
– immer	14 (6,8)	14 (19,2)	0 (0,0)	< 0,001
– häufig	52 (25,4)	46 (63,0)	6 (4,5)	
– selten	65 (31,7)	13 (17,8)	52 (39,4)	
– nie	74 (36,1)	0 (0,0)	74 (56,1)	
Wie sicher fühlen Sie sich in der palliativen Betreuung von Menschen mit fortgeschrittener Herzinsuffizienz?				
– sehr sicher	93 (46,3)	36 (52,2)	57 (43,2)	0,07
– eher sicher	66 (32,8)	24 (34,8)	42 (31,8)	
– eher unsicher	30 (14,9)	4 (5,8)	26 (19,7)	
– sehr unsicher	12 (6,0)	5 (7,2)	7 (5,3)	
Ich habe bei Patient:innen selbst antitachykarde Funktionen implantierter Devices deaktiviert				
– immer	0 (0,0)	0 (0,0)	0 (0,0)	< 0,001
– häufig	3 (1,4)	0 (0,0)	3 (2,2)	
– selten	96 (45,5)	4 (5,2)	92 (68,7)	
– nie	112 (53,1)	73 (94,8)	39 (29,1)	
Aus welchen Gründen (Mehrfachauswahl möglich)				
– auf Wunsch der Patientin/des Patienten	81 (82,7)	1 (33,3)	80 (84,2)	0,129
– um rezidivierenden Schocks zuvorzukommen	67 (68,4)	2 (66,7)	65 (68,4)	1
– auf Anregung von Angehörigen	31 (31,6)	1 (33,3)	30 (31,6)	1
– nach rezidivierenden Schocks, die zu Leidensdruck führen	25 (25,5)	1 (33,3)	24 (25,3)	1

Die Kategorie „Niedergelassene in der hausärztlichen Versorgung“ beinhaltet Fachärzt:innen für Allgemeinmedizin und Fachärzt:innen für Innere Medizin in der hausärztlichen Versorgung. Subgruppen wurden mittels Chi-Quadrat-Test verglichen

Insgesamt fühlte sich die Mehrzahl der befragten Ärzt:innen sehr sicher (46,3 %) oder eher sicher (32,8 %) in der palliativen Betreuung von Patient:innen mit schwerer Herzinsuffizienz (kein signifikanter Unterschied zwischen Hausärzt:innen und niedergelassenen Kardiolog:innen).

### Zusammenarbeit mit der SAPV

Die Mehrzahl der befragten Ärzt:innen gab an, dass ihre Patient:innen mit fortgeschrittener Herzinsuffizienz selten (58,9 %) oder nie (13,0 %) durch SAPV mitbehandelt wurden. Hausärzt:innen gaben signifikant häufiger an, selbst eine SAPV für Patientinnen mit fortgeschrittener Herzinsuffizienz verordnet zu haben (78,4 % vs. 31,3 %; *p* < 0,001). Die häufigsten Gründe für die Initiierung einer SAPV-Mitbehandlung waren die Gewährleistung der pflegerischen Versorgung in der Häuslichkeit (85,9 %) sowie die Vermeidung von Hospitalisierungen in der letzten Lebensphase (77,8 %). Als Problem bei der Initiierung der SAPV-Betreuung wurde am häufigsten das Fehlen zeitlicher Ressourcen angegeben (67,7 %). Seltener wurden als Probleme die fehlende Ausrichtung von SAPV-Dienstleistungen auf die Bedürfnisse von Herzinsuffizienzpatient:innen (33,3 %), fehlende kardiologische Expertise von SAPV-Dienstleistern (17,4 %), Ablehnung durch SAPV-Dienste (16,4 %) oder Ablehnung durch Kostenträger (11,3 %) angegeben. Mehr Informationen hierzu finden sich in Tab. 4.

**Tab. 4:** Erfahrungen mit der Spezialisierten ambulanten Palliativversorgung (*SAPV*)

	Insgesamt	Niedergelassene in der hausärztlichen Versorgung	Niedergelassene in der Kardiologie	*p*
	*n* = 235	*n* = 89	*n* = 146	
Meine Patient:innen mit fortgeschrittener Herzinsuffizienz wurden durch Einrichtungen der SAPV (Palliativstation, Hospiz) (mit)betreut				
– immer	4 (1,9)	3 (4,0)	1 (0,8)	0,025
– häufig	54 (26,1)	27 (36,0)	27 (20,5)	
– selten	122 (58,9)	37 (49,3)	85 (64,4)	
– nie	27 (13,0)	8 (10,7)	19 (14,4)	
Auf welche Art und Weise (Mehrfachauswahl möglich)				
– SAPV-Team	125 (69,8)	63 (94,0)	62 (55,4)	< 0,001
– Palliativstation	85 (47,5)	26 (38,8)	59 (52,7)	0,1
– Hospiz	87 (48,6)	25 (37,3)	62 (55,4)	0,029
Ich habe Patient:innen mit fortgeschrittener Herzinsuffizienz selbst in Einrichtungen der SAPV angemeldet				
– immer	6 (2,9)	6 (8,1)	0 (0,0)	< 0,001
– häufig	20 (9,8)	20 (27,0)	0 (0,0)	
– selten	73 (35,6)	32 (43,2)	41 (31,3)	
– nie	106 (51,7)	16 (21,6)	90 (68,7)	
Was waren für Sie Gründe, die spezialisierte Palliativmedizin hinzuzuziehen? (Mehrfachauswahl möglich)				
– Gewährleistung einer ausreichenden pflegerischen Versorgung in der Häuslichkeit	85 (85,9)	48 (82,8)	37 (90,2)	0,447
– Vermeidung von Hospitalisationen	77 (77,8)	46 (79,3)	31 (75,6)	0,849
– Sterbebegleitung	68 (68,7)	36 (62,1)	32 (78,0)	0,142
– Eindosierung von Opioiden zur Symptomkontrolle	33 (33,3)	15 (25,9)	18 (43,9)	0,097
– Unterstützung bei psychischem oder spirituellem Distress	28 (28,3)	17 (29,3)	11 (26,8)	0,965
Welche Probleme in Bezug auf die palliativmedizinische Versorgung von Patient:innen mit fortgeschrittener Herzinsuffizienz sind Ihnen in Ihrer praktischen Arbeit begegnet? (Mehrfachauswahl möglich)				
– Meine Zeit im Rahmen der Sprechstunde hat nicht ausgereicht, um dem palliativmedizinischen Bedarf meiner Patient:innen gerecht zu werden	132 (67,7)	40 (59,7)	92 (71,9)	0,118
– Palliativmedizinische Konzepte waren nicht ausreichend auf Bedürfnisse kardiologischer Patient:innen ausgerichtet	65 (33,3)	24 (35,8)	41 (32,0)	0,709
– Palliativmediziner:innen hatten zu wenig kardiologische Expertise	34 (17,4)	5 (7,5)	29 (22,7)	0,014
– Spezialisierte Palliativeinrichtungen (Hospiz, Palliativstation, SAPV-Team) lehnte Patient:innen ab	32 (16,4)	15 (22,4)	17 (13,3)	0,154
– Kostenträger lehnten die palliativmedizinische Versorgung ab	22 (11,3)	12 (17,9)	10 (7,8)	0,06

Die Kategorie „Niedergelassene in der hausärztlichen Versorgung“ beinhaltet Fachärzt:innen für Allgemeinmedizin und Fachärzt:innen für Innere Medizin in der hausärztlichen Versorgung. Subgruppen wurden mittels Chi-Quadrat-Test verglichen

## Diskussion

Der Großteil der befragten niedergelassenen Ärzt:innen in Deutschland behandelt Menschen mit fortgeschrittener Herzinsuffizienz. Kommunikation über Prognose und Therapieziele wird von nur etwas mehr als der Hälfte der Befragten durchgeführt, tendenziell im fortgeschrittenen Krankheitsverlauf, wenn Ärzt:innen bereits eine Verschlechterung des klinischen Zustands feststellen. Die palliative Grundversorgung, z. B. durch ganzheitliches Symptommanagement, Mitbehandlung psychischer Probleme und Unterstützung bei der Optimierung der häuslichen Versorgungssituation und Sterbebegleitung, wird häufig von Hausärzt:innen, hingegen eher selten von niedergelassenen Kardiolog:innen übernommen, die in der überwiegenden Mehrzahl Patient:innen nicht mehr weiterbetreuen, wenn diese nicht mehr in der Lage sind, in ihre Praxis zu kommen. SAPV wird bei fortgeschrittener Herzinsuffizienz insgesamt eher selten verordnet und wenn, dann häufiger durch Hausärzt:innen als durch Kardiolog:innen.

### Kontextualisierung der Studienergebnisse

Unsere Studienergebnisse müssen im Kontext des Studiendesigns und der damit einhergehenden Limitationen interpretiert werden. Nach Informationen des BNK e. V. sind in Deutschland rund 1300 Kardiolog:innen in etwa 430 Praxen niedergelassen. Da die teilnehmenden Kardiolog:innen dieser Studie jeweils eigene Praxen repräsentieren, bildet unsere Umfrage ein Viertel bis ein Drittel aller kardiologischen Praxen in Deutschland ab und hat damit einen ernstzunehmenden Stichprobenumfang. Die Repräsentativität der Hausärztestichprobe dieser Studie ist schwieriger einzuschätzen und dürfte niedriger liegen. Unsere Umfrage erfasst die Selbsteinschätzung niedergelassener Ärzt:innen in Bezug auf ihr eigenes ärztliches Handeln. Die tatsächliche Behandlungsrealität könnte von dieser Selbsteinschätzung durchaus abweichen und kann in unserem Studiendesign nicht direkt erfasst werden. Die soziale Erwünschtheit ganzheitlichen ärztlichen Handelns könnte dazu geführt haben, dass Befragte ihr eigenes palliativmedizinisches Handeln überschätzt haben. Zudem könnte Selbstselektion bei der Entscheidung zur Teilnahme an dieser Onlineumfrage einen systematischen Bias in unseren Daten bedingen: Basierend auf der Annahme, dass Niedergelassene mit Interesse an und Erfahrung in palliativer Versorgung mit höherer Wahrscheinlichkeit an dieser Umfrage teilgenommen haben, müssen wir davon ausgehen, dass in der Grundgesamtheit der Niedergelassenen in Deutschland palliative Versorgung bei Herzinsuffizienz schlechter integriert ist, als unsere Ergebnisse annehmen lassen.

### Bestehende Literatur zur ambulanten Palliativversorgung bei Herzinsuffizienz

Es gibt kaum systematische Erhebungen zur ambulanten palliativen Versorgungsituation bei Herzinsuffizienz in Deutschland. In einer Auswertung von SAPV-Daten der Kassenärztlichen Vereinigung Nordrhein war Herzinsuffizienz in nur 4 % aller Fälle die Indikation für eine SAPV-Versordnung. Mit einer medianen Verweildauer in SAPV von 9 Tagen wurden die wenigen Herzinsuffizienzpatient:innen, die überhaupt in die SAPV eingeschlossen wurden, erst sehr kurz vor dem Lebensende überwiesen [9]. Unsere Studienergebnisse bestätigen, dass SAPV für Menschen mit fortgeschrittener Herzinsuffizienz bislang eher eine Ausnahme darstellt. Grund hierfür scheinen nicht Ablehnung durch Kostenträger oder SAPV-Dienste oder mangelnde Kompetenzen auf SAPV-Seite, sondern eher organisatorische Hürden, wie z. B. Zeitmangel auf Seite der Niedergelassenen zu sein.

Unsere Studienergebnisse zeigen, dass die palliative Grundversorgung im Sinne einer multidimensionalen Erfassung physischer und psychosozialer Probleme eher von Hausärzt:innen als von niedergelassenen Kardiolog:innen übernommen wird. Dies entspricht der Maßgabe der Nationalen Versorgungsleitlinie Herzinsuffizienz (NVL), die klar feststellt: „Hauptträger der allgemeinen Palliativversorgung sind die Hausärzte.“ Diese seien auch dafür zuständig, Patient:innen mit SAPV-Bedarf zu identifizieren und entsprechend weiterzuvermitteln [7]. Welche Rolle niedergelassene Kardiolog:innen in der ambulanten Palliativversorgung von Menschen mit fortgeschrittener Herzinsuffizienz einnehmen sollten, bleibt in der NVL undefiniert.

Qualitative Studien haben gezeigt, dass insbesondere Kardiolog:innen zwar grundsätzlich offen für palliative Versorgung sind, jedoch meist wenig inhaltliches Wissen über palliative Versorgung haben [10–12]. Auch wir konnten eine gewisse kognitive Dissonanz dokumentieren: Ein Großteil der befragten niedergelassenen Kardiolog:innen gab an, sich sehr sicher in palliativer Versorgung zu fühlen – nur wenige hingegen gaben an, konkret auch palliative Bedürfnisse zu behandeln und zu adressieren.

Eine kontinuierlich wachsende Zahl prospektiver Studien befasst sich mit der Effektivität palliativmedizinischer Interventionen bei Herzinsuffizienz, tendenziell mit positiven Effekten auf die Lebensqualität [13–17]. Der Early Palliative Care in Heart Failure (EPCHF) Trial lieferte zum ersten Mal auch prospektiv randomisierte Ergebnisse aus Deutschland: Regelmäßige Nachsorgetermine in einer palliativmedizinischen Hochschulambulanz hatten dabei keinen Effekt auf die Lebensqualität einer unselektierten Kohorte von Herzinsuffizienzpatient:innen. Hierzu könnte die fehlende Patientenselektion beigetragen haben [18, 19]. Studien, die die Effektivität von allgemeiner ambulanter Palliativversorgung durch Niedergelassene oder aufsuchenden ambulanten Palliativangeboten wie SAPV in Deutschland untersuchen, liegen nicht vor; hier besteht weiterer Forschungsbedarf.

### Implikationen für die Zukunft

Während die NVL Herzinsuffizienz für Deutschland die palliative Versorgung bei fortgeschrittener Herzinsuffizienz grundsätzlich empfiehlt, bleibt die praktische Umsetzung größtenteils unkonkretisiert. Es gibt bislang kaum evidenzbasierte Handlungsempfehlungen dazu, wie Symptome ganzheitlich erfasst und leitliniengerecht behandelt werden sollten, wie Opioide wirksam eingesetzt werden können oder welche Rolle die krankheitsgerichtete Pharmakotherapie für die Symptomkontrolle spielt, welche Patient:innengruppen einer SAPV zugeführt werden sollten sowie wie strukturiert Gespräche geführt, Therapieziele festgelegt und Therapieentscheidungen getroffen werden können. Oft muss Evidenz aus Studien an Patient:innen mit Tumorerkrankungen auf die Herzinsuffizienzpopulation extrapoliert werden [7]. Hier sind sowohl Fachgesellschaften in der Erstellung praktischer Handlungsempfehlungen für alle Professionen als auch Forschende in der Beantwortung offener Forschungsfragen gefragt.

Nebenbei konnten wir dokumentieren, dass die große Mehrzahl der niedergelassenen Kardiolog:innen ihre Patient:innen nicht mehr weiterbehandelt, wenn diese nicht mehr in die Praxis kommen können. Dies erschwert den Zugang zu fachkardiologischer Betreuung genau für Patient:innen mit weit fortgeschrittener Herzinsuffizienz oder schlechter Gesamtkonstitution, deren kardiovaskuläre Behandlung besonders herausfordernd ist. Außerdem scheint die Deaktivierung implantierter Devices durch niedergelassene Kardiolog:innen nur selten stattzufinden. Dies steht im klaren Kontrast zur hohen Prävalenz dieser Therapien in Deutschland. Systematische Erhebungen zur Anzahl der Device-Deaktivierungen in Deutschland sind uns nicht bekannt. Zur Bewältigung dieser Herausforderung bedarf es der weiteren Erforschung und Weiterentwicklung bestehender Versorgungsformen auf systemischer Ebene, aber auch der Sensibilisierung aller klinisch Handelnden, um den komplexen Bedürfnissen von Patient:innen mit fortgeschrittener Herzinsuffizienz in Zukunft besser gerecht werden zu können.

## Fazit für die Praxis

- Nur wenige Niedergelassene in Deutschland überweisen ihre Patient:innen mit fortgeschrittener Herzinsuffizienz regelmäßig in spezialisierte palliativmedizinische Versorgungsstrukturen.
- Ambulante palliative Grundversorgung und die Involvierung der spezialisierten ambulanten Palliativversorgung (SAPV) werden sehr viel häufiger von Hausärzt:innen als von niedergelassenen Kardiolog:innen übernommen.
- Weitere Initiativen zu einer besseren Erfassung der Versorgungsrealität in Deutschland wären wünschenswert.

## Data Availability

Studiendaten können beim korrespondierenden Autor angefragt werden.
